# Analysis of Historical Trends and Recent Elimination of Malaria from Sri Lanka and Its Applicability for Malaria Control in Other Countries

**DOI:** 10.3389/fpubh.2017.00212

**Published:** 2017-08-28

**Authors:** Dilkushi Anula Wijesundere, Ranjan Ramasamy

**Affiliations:** ^1^Asiri Hospital, Colombo, Sri Lanka; ^2^ID-FISH Technology, Palo Alto, CA, United States

**Keywords:** global malaria eradication, malaria, malaria elimination, malaria transmission rates, Sri Lanka, vector control, zoonotic malaria

## Abstract

Sri Lanka is a tropical island located South of India in the Indian Ocean. Malaria has been prevalent in the island for centuries but the country succeeded in eliminating the disease in 2013. Factors governing the past endemicity of malaria and its successful elimination from Sri Lanka in 2013 are analyzed. There is evidence that malaria might have been first introduced in the thirteenth century into a dry zone area with extensive irrigation works. Regular widespread epidemics of the disease have been documented in the twentieth century. The island nature of Sri Lanka, generally low transmission rates, widespread and accessible government hospitals and clinics that provide free and readily available diagnosis and treatment for malaria, adequate financial support and commitment to the Antimalaria Campaign (AMC), national and decentralized malaria control efforts sustained over a long period by dedicated and competent AMC staff, and the absence of zoonotic malaria are recognized as key factors responsible for eliminating malaria from Sri Lanka. These factors are analyzed in the context of their relevance to the present malaria elimination efforts in other countries with the overall aim of globally eradicating the disease.

## Current Global Malaria Situation

Malaria is one of the most widespread parasitic diseases in the world. It is caused by four species of protozoan parasites that are transmitted between humans by anopheline mosquitoes, namely *Plasmodium falciparum, Plasmodium vivax, Plasmodium malariae*, and *Plasmodium ovale*, as well as different species of zoonotic malaria parasites, e.g., *Plasmodium knowlesi* (1). Globally an estimated 3.2 billion people in 95 countries are presently at risk of infection with malaria (2).

According to the most recent available WHO estimate (2), there were 212 million new cases of malaria worldwide in 2015 (range 148 million to 304 million). The African region accounted for the majority of global cases of malaria (90%) followed by the Southeast Asian (7%) and Eastern Mediterranean (2%) regions. Such high endemicity resulted in an estimated 429,000 deaths (range 235,000–639,000) worldwide. Out of these deaths, 306,000 were of children under the age of 5 years. Most deaths in 2015 are estimated to have occurred in the WHO African region (92%), followed by the WHO Southeast Asia region (6%) and the WHO Eastern Mediterranean region (2%). Malaria due to *P. falciparum* caused 99% of the deaths from malaria making it, by far, the most lethal of the human-infective malaria parasites. These data from the WHO (2) confirm the magnitude of the current malaria situation and its consequences for human well-being in the global context.

The WHO has developed a set of targets to be attained by 2030 in a Global Technical Strategy for Malaria 2016–2030 (3). These are: by 2030, to reduce malaria incidence and mortality rates globally by at least 90% compared with 2015 levels; to eliminate malaria from at least 35 countries in which malaria was transmitted in 2015; and to prevent re-establishment of malaria in all countries that are malaria free. These WHO targets are designed to fulfill a target set by the relevant United Nations Sustainable Development Goal, which was to end the epidemics of AIDS, tuberculosis, malaria, and neglected tropical diseases by 2030 (4).

## History of Malaria in Sri Lanka

**Figure 1:**
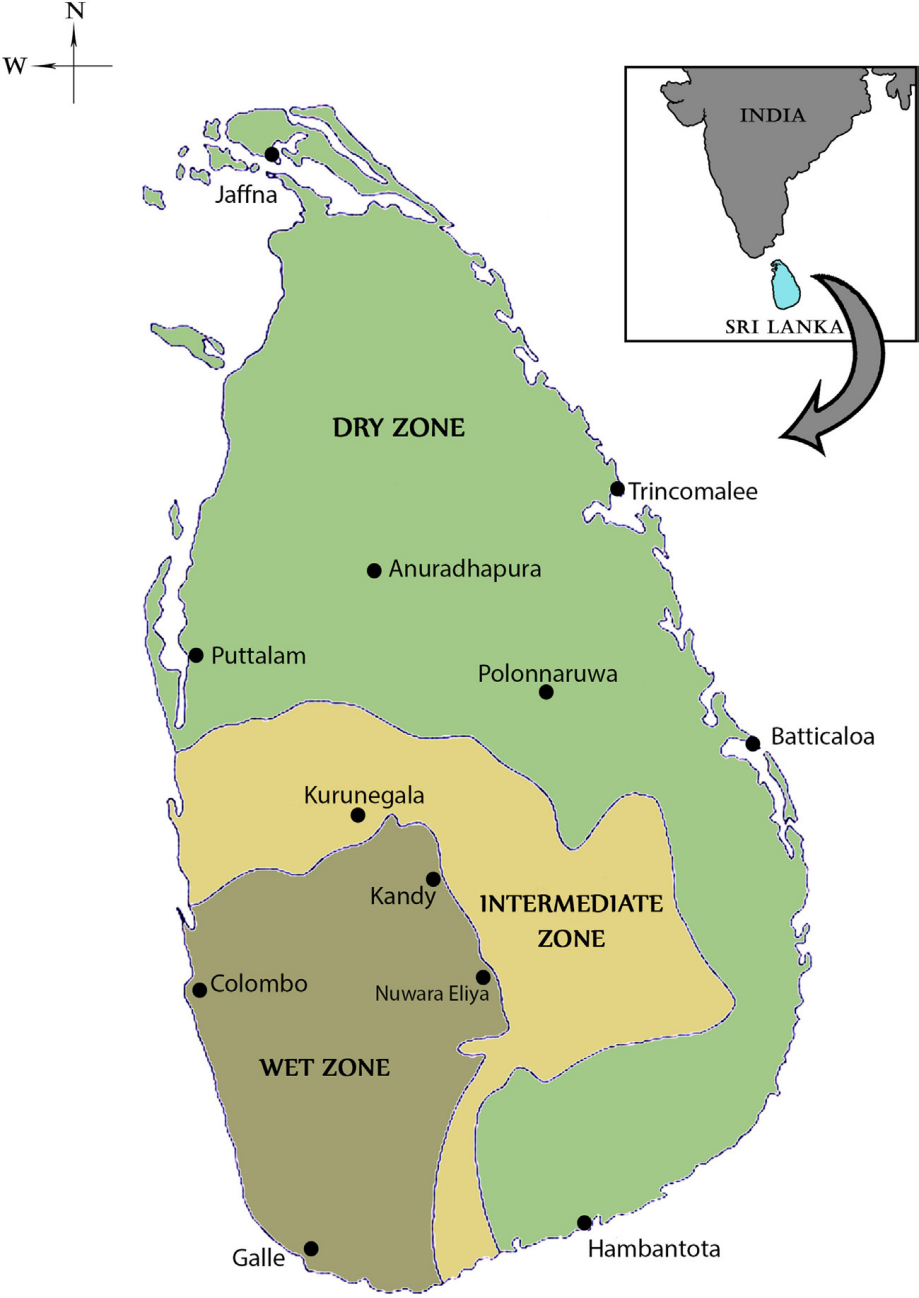
Map of Sri Lanka showing the dry, wet, and intermediate rainfall zones. The locations of major cities, including the ancient cities of Anuradhapura and Polonnaruwa are shown in the map.

Sri Lanka is an island with an area of 65,525 km^2^ located between latitudes 5′55 and 9′50 north of the equator (Figure 1). It lies in close proximity to southern India and the two countries are separated by the 64–137 km wide Palk Strait. The central hills of the island divide the surrounding plains into dry and wet rainfall zones. The wet zone, located in the central hills and the Southwest of the island, receives an average annual rainfall of 250 cm in two main rainy seasons, namely the Northeast (NE) monsoon that typically commences in October and ends in January, and the Southwest (SW) monsoon that often begins in April and ends in June. Inter-monsoonal rains also occur between these periods in the wet zone. The dry zone, with an annual rainfall of 60–190 cm, receives maximal rainfall during the NE monsoon and typically little or no rain for the rest of the year. An intermediate zone, with mixed characteristics, lies between the dry and wet zones. Malaria was typically endemic in the dry and intermediate rainfall zones of the country with the wet zone becoming prone to malaria during exceptional dry conditions caused by a failure of the monsoons (5–11). Only the high hill country is free from malaria transmission because of the cold (5–11).

**Figure 2:**
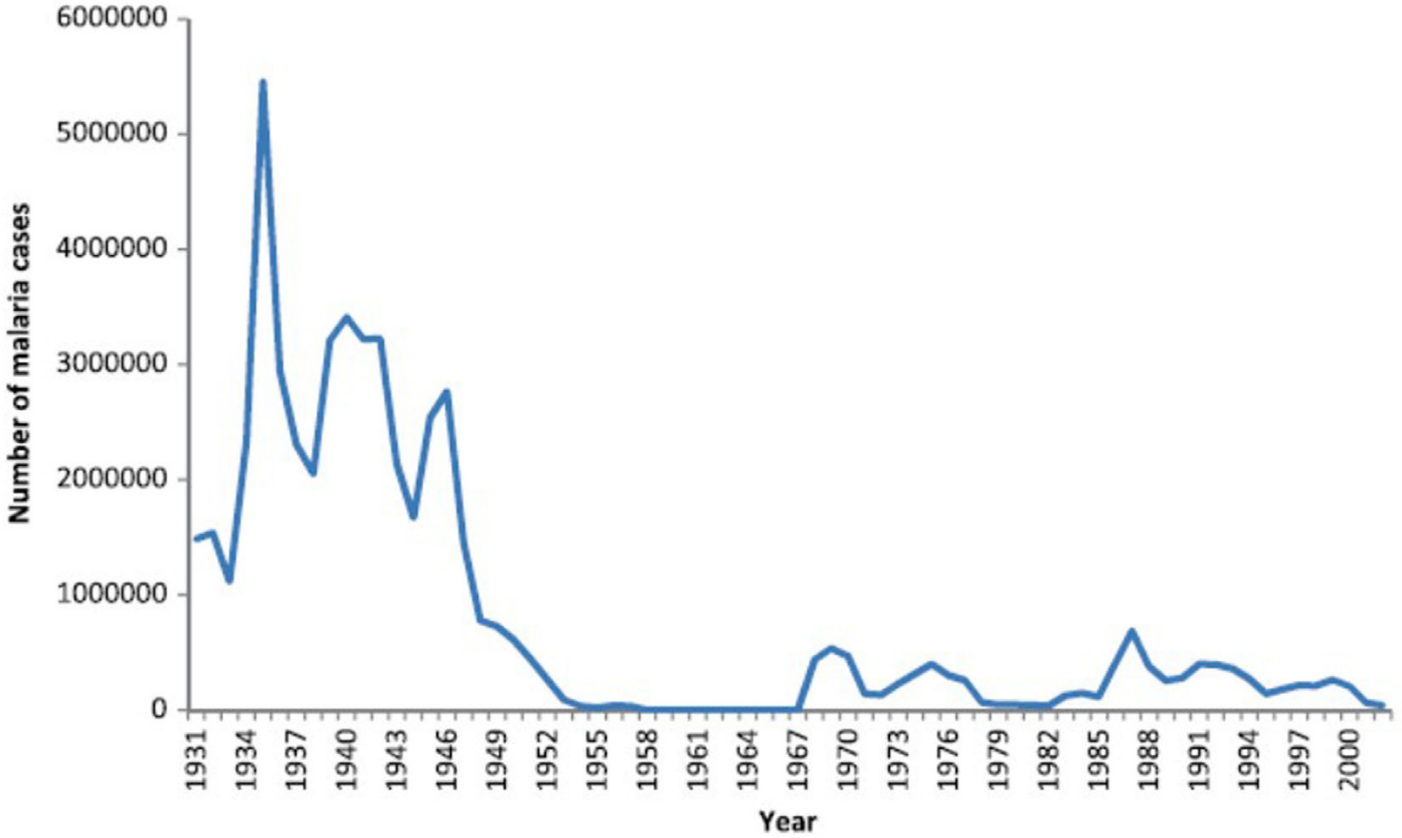
Graph showing the prevalence of malaria in Sri Lanka from 1931 to 2000. Reproduced from Karunaweera et al. Malaria Journal (2014) 13:59 under the creative commons license (13).

There were no written records of a malaria-like disease in Sri Lanka until the early thirteenth century when such a disease was associated with the decline of Polonnaruwa Kingdom (1029–1232 AD) as recorded by Buddhist monks in the chronicle Mahavamsa. There was no such record of malaria in the preceding Anuradhapura kingdom (544 BC–1029 AD) that, like Polonnaruwa, was located in the present day North Central province in the dry zone (Figure 1). The Polonnaruwa kingdom had a typical dry zone ecology. The kings of that time conceived the idea of harnessing water from the great rivers flowing into the region originating in the central hills of the island and supplied by rainfall from both monsoons (12). Dams were built and canals constructed to conduct water toward the major areas of rice cultivation in the present day North Central province. There, the water was stored in large irrigation reservoirs. The ancient hydraulic systems of dams, canals, sluices, and reservoirs were built to a high engineering standard. The agricultural success resulting in the irrigation development during the Anuradhapura and Polonnaruwa era spawned a thriving civilization that became famous for flourishing Buddhist cultural and literary activities, arts and crafts, and the architectural wonders of temples. The collapse of the Polonnaruwa kingdom in the thirteenth century has been variously associated with invasions from South India and epidemics of the malaria-like disease (12). The malaria-like disease appears to have continued to persist in the dry zone after the decline of Polonnaruwa. A subsequent record of a malaria-like disease comes from a map published by the Dutch during the period of Dutch colonization of the island, 1658–1815. This indicated that the Southern Province located in the wet zone of the island had been depopulated by a febrile illness, which had also occurred in the dry zone areas surrounding Polonnaruwa. Malaria was conjectured to be the scourge and, by the 1880s, it was identified as “Kale Una” or “Forest Fever” by the affected Sinhalese communities. Malaria was firmly established in Sri Lanka by the beginning of the twentieth century and caused serious epidemics in 1906, 1914, 1919, 1923, 1934–1935, 1967–1969, and 1986–1987 (13, 14) (Figure 2).

The country’s first major effort to control malaria began with the establishment of the Antimalaria Campaign (AMC) by the government in 1911 during the British Colonial period (1815–1948). The country’s first malariologist was appointed in 1921. In the early 1920s, different approaches were used in urban and rural areas to control malaria. Antilarval measures [e.g., application of Paris green or copper (II) acetate triarsenite] were used in rural areas while environmental measures (drainage and filling of small pools and water collections) proved cost effective in urban areas. However, in rural areas with an abundance of potential breeding sites, these measures were relatively infective and malaria spread rapidly reaching a major epidemic proportion in 1934–1935.

## The Great Malaria Epidemic of 1934–1935

The most devastating of all AMC-recorded malaria epidemics in Sri Lanka was the epidemic of 1934–1935, which ravaged the entire island, infecting an estimated five million people and causing approximately 80,000 deaths (15). *Plasmodium vivax* was the predominant parasite species being responsible for >50% of infections during this epidemic, but there was also a significant and time-variable contribution from *P. falciparum* (up to 43%) and a relatively minor contribution (approximately 1%) from *P. malariae* (15). Almost one out of every two infants born during this epidemic died of malaria. The devastating impact of this epidemic in the North Central province is shown in Table 1. The number of cases of malaria was determined by blood film examination at this time.

**Table 1:** The malaria epidemic of 1934–1935 in the North Central Province.

	1934	1935
Number of cases in the province	108,857	194,144
Death rate per 1,000 population	36.1	87.5
Maternal mortality rate per 1,000 live births	27.2	50.5
Infant mortality rate per 1,000 live births	347.5	481

*Data from the Ceylon Government Administrative Report of the North Central Province, 1935*.

In order to bring the epidemic under control, larval habitats were cleared, drained, and filled to prevent stagnant water collection and oiling was carried out to minimize preimaginal development of the anopheline vectors in other water bodies. Quinine was widely used for therapy during this epidemic for treating patients (15). The medical authorities attributed the main cause of the epidemic to an unusually prolonged drought in the whole country that led to drying up of the major rivers resulting in the formation of riverine pools that provided expanded and ideal habitats for preimaginal development of *Anopheles culicifacies*, the major malaria vector in the country (15). However, population movements may also have been a factor for this epidemic. Due to a prevailing economic depression and scarcity of employment, persons working in the wet zone coconut and rubber plantations migrated to the dry zone, where they contracted malaria within a short time and then returned to their former homes because of their illness. Such people would have been important carriers for introducing the parasite into the wet zone where anopheline densities were also increased by the prevailing drought conditions.

## Malaria in Sri Lanka During the Second Half of the Twentieth Century

Dichlorodiphenyltrichloroethane (DDT), an organochlorine insecticide, was introduced in Sri Lanka after World War 2 for use as an indoor residual insecticidal spray to control malaria. The use of DDT spraying at this time was highly effective and led to the decline of malaria in many countries including Sri Lanka. Consequently, the WHO declared the global eradication of malaria to be a goal in 1955 and Sri Lanka became a pioneering nation in launching its own malaria eradication program in 1958. With its incidence dropping rapidly in the country, malaria was made a notifiable disease in 1961. After years of malaria control by the AMC, the number of cases in the whole island rapidly declined to only 6 indigenously transmitted and 11 imported cases of malaria in 1963 (13). For the first time in recorded history, malaria was on the brink of elimination in Sri Lanka.

With elimination around the corner, Sri Lankan government authorities became complacent and made a disastrous mistake. Continuous vigilance was not maintained and control measures were reduced. Many DDT spraying teams were disbanded and a consolidation phase of eradication (as defined by the WHO) was initiated in 1964 (14, 16). Because of the relaxed malaria control measures, a gradual increase in malaria incidence occurred from 1965 onward, leading to a countrywide epidemic of malaria in 1967–1968 with over 500,000 malaria cases being reported in 1969 (13). The return of malaria led to a resumption of vector control measures, principally the indoor residual spraying of insecticides, by the AMC. Fortunately and surprisingly, only five deaths were reported as being due to malaria in 1969. This may have been because 90% of the parasite cause of the epidemic was *P. vivax*, a less fatal parasite species *P. falciparum*, and for which chloroquine treatment had then become readily available in all parts of the country under the widespread and free government health service. The ensuing years showed many small peaks of malaria incidence. The principal vector of malaria in the island, *An. culicifacies*, had begun to develop resistance to DDT by 1969. Due to increasing vector resistance to DDT, it was replaced by the organophosphate insecticide Malathion for vector control by indoor residual spraying in 1977.

## Resurgence of Malaria 1980–2000

These two decades saw the establishment of many new irrigation schemes throughout the country, especially in the malaria endemic areas of the dry zone. Despite the widespread use of Malathion, malaria continued to be prevalent with high incidence from 1980 to 2000 (Figure 2). The most important irrigation scheme undertaken and established during this period was the accelerated Mahaweli Development Scheme. The Mahaweli, the country’s largest river, was dammed and large storage reservoirs and canals built to divert the water into areas of rice cultivation in the North Central Province. This large project included the Victoria, Kothmale, Polgolla, Randenigala, Rantambe, and Maduru Oya sub-projects situated in the central hill country. With the establishment of these reservoirs in the hill country, several towns here were inundated by the Polgolla and Victoria reservoirs. Many people of the hill country who lost their homes due to the Mahaweli Development Scheme were translocated to the newly established agricultural areas of the North Central Province in System B of the Mahaweli project within the Polonnaruwa district (6). This process led to mixing of people who had lived in the Polonnaruwa district for decades and developed a degree of acquired clinical immunity to malaria, with the new colonists from non-endemic areas in the hill country with no acquired immunity to malaria. When the non-immune new arrivals became infected with malaria, they developed very high parasitemia contributing to malaria transmission in the population.

Malaria reached epidemic proportions in System B of the Mahaweli project in the period 1986–1988 (10). Many inhabitants became seriously ill with malaria for the first time during the epidemic in 1986. Apart from changes in ecology and population structure, failure of vector control was also considered to have contributed to this epidemic. Inadequate spraying of Malathion resulted from the major fire at the Malathion storage plant in 1986, which destroyed much of the imported chemical. Furthermore, pilferage of Malathion from stores (to be used as agricultural insecticide) and decreased acceptance of Malathion spraying due to its pungent odor were also causative. The civil war in parts of the Northern and Eastern province at this time also had an impact on indoor residual spraying and other vector control activities (17). Reservoirs of poorly controlled vectors in conflict zones may have contributed to an increase in malaria transmission in the contiguous Polonnaruwa district in the North Central province.

## Malaria Epidemic in Polonnaruwa 1986–1987

The most significant feature of the malaria epidemic at this time was the tremendous surge of *P. falciparum* malaria. *P. vivax* had previously been recorded as the predominant parasite species causing malaria in Sri Lanka by the AMC since 1911. However, the *P. falciparum: P. vivax* case ratio reached nearly 1:1 for the first time during this epidemic as illustrated by the data for the Polonnaruwa district in Table 2. However, this proportionate increase in *P. falciparum*, which was also reflected in other parts of the country, later reversed closer to the year 2000 and afterward (see below).

**Table 2:** Malaria case numbers for the Polonnaruwa district.

	1972	1980	1985	1987
*Plasmodium vivax*	7,850	1,789	1,955	13,849
*Plasmodium falciparum*	13	27	496	12,554

*Data from the Anti Malaria Campaign, Ministry of Health, Sri Lanka*.

The high prevalence of malaria in the Polonnaruwa district was further confirmed by the assessment of the spleen rate, the parasite rate, and the pattern of malarial admissions to Base Hospital, Polonnaruwa. We observed a high prevalence of anemia, splenomegaly, hepatomegaly, and fever with attendant other complications of malaria at this hospital during this period (18, 19). Guillain–Barre syndrome was also first described as a complication of malaria at this time (20).

Our own observations in Weheragala, a village located within the Polonnaruwa district in system B of the Mahaweli irrigation scheme, showed a continued high prevalence of malaria in the area in 1990 and generated pertinent information on its possible cause (6). At Weheragala, in 1990, up to 72, 59, 40, and 20.2% of the inhabitants had anemia, splenomegaly, hepatomegaly, and positive blood films for malaria parasites, respectively. The *P. falciparum* to *P. vivax* ratio varied from 1.5 to 0.5 in blood films (6). We observed an exceptionally high entomological inoculation rate of 0.12 infective bites per person per hour with *Anopheles annularis*, previously recognized only as a minor malaria vector in Sri Lanka (6). This was because 93% of all anophelines collected in the village proved to be *An. annularis*, a mosquito species, which adapts well to lay eggs and undergo larval development in small irrigation channels. Later, molecular biological studies showed that the *An. annularis* in Sri Lanka was sibling species A, which is also characteristically known to develop in riverine and water channel habitats in India (21). We proposed that *An. annularis*, and other anopheline vectors such as *An. culicifacies*, which was also found in Weheragala, could serve to maintain a high level of malaria transmission in the vicinity of new irrigation projects and thereby form nuclei of infections for producing malaria epidemics throughout the country (6). One can reasonably speculate that malaria may have been introduced into ancient Polonnaruwa by infected persons arriving from other malaria endemic nations and then spread rapidly because of similar conducive conditions, namely a high density of potential vectors that developed in the vast irrigation schemes constructed by the Polonnaruwa kings. There was also no effective drug treatment for malaria at that time. Malaria could, therefore, have led to the rapid depopulation of Polonnaruwa and other ancient towns and cities that were dependent on dry zone irrigation schemes for their sustenance.

## Landmark Developments in Malaria Control in the 1990s in Sri Lanka

A significant development in malaria control in Sri Lanka in the 1990s was the devolvement of many of the responsibilities of the Antimalaria Campaign of the Ministry of Health to the provincial Ministries of Health in 1991. This allowed control measures to be applied to better suit local conditions and needs. Vector control activities were maintained at varying levels of effectiveness by the decentralized services even in areas affected by the civil war (17).

Another important development was the discontinuance of Malathion for indoor residual spraying in 1993 in most parts of the country as a result of the development of resistance to it among the major vectors (22). Malathion was replaced with λ-cyhalothrin, a synthetic pyrethroid. Insecticides such as fenitrothion, λ-cyhalothrin, cyfluthrin, deltamethrin, and etofenprox were used in different districts on a rotational basis to delay the development of resistance in mosquitoes (22). At the same time, long-lasting insecticide (pyrethroid)-treated nets began to be distributed by the Anti Malaria Campaign as a supplementary malaria control measure. However, Malathion continued to be used in the war-affected northern and eastern provinces until toward the end of the civil war in 2009 when it was replaced by the more effective pyrethroid insecticides.

## Malaria after the Year 2000 in Sri Lanka

**Figure 3:**
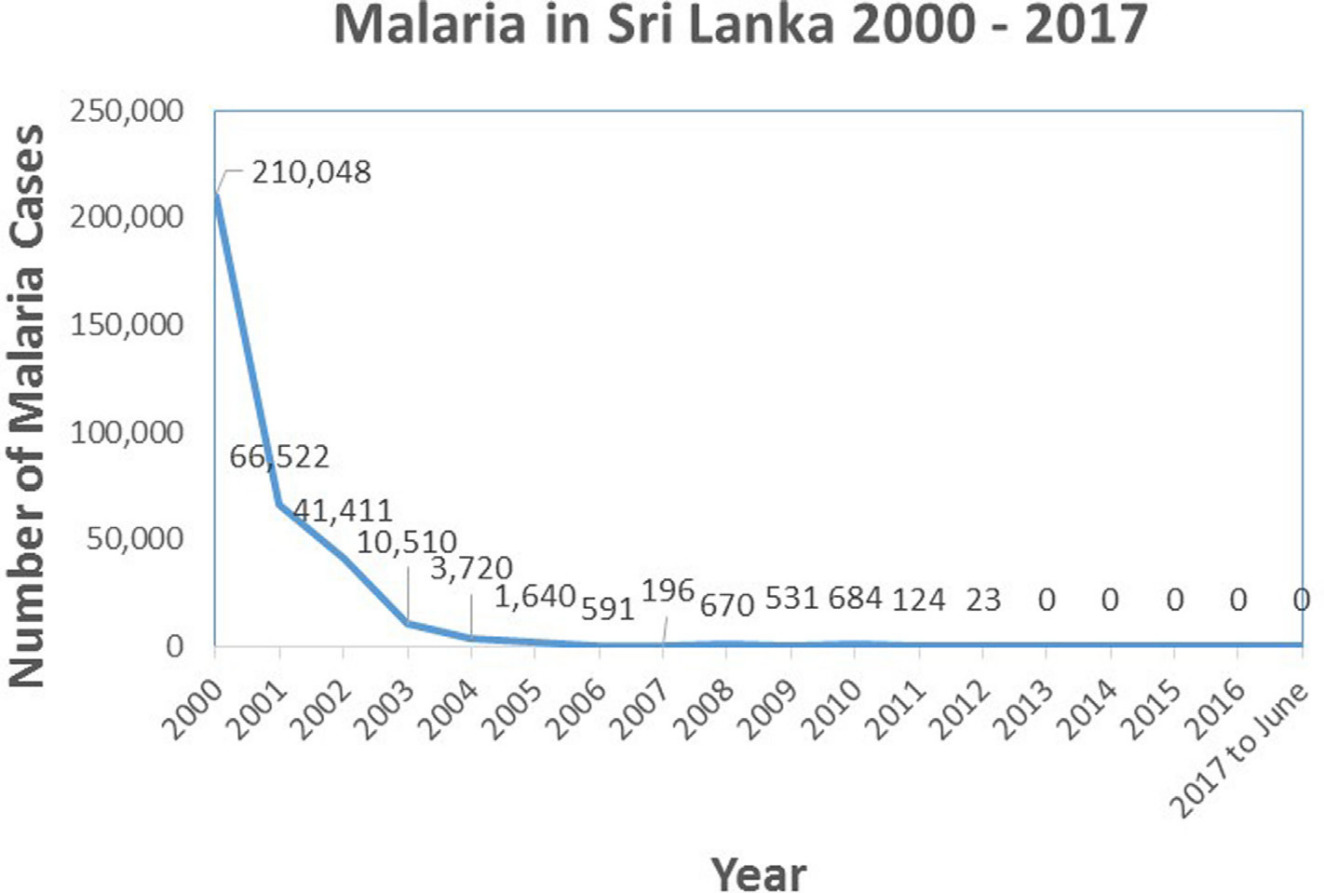
Graph showing the prevalence of indigenously transmitted cases of malaria in Sri Lanka from the year 2000 until June 2017. Source of data: Antimalaria Campaign, Ministry of Health, Sri Lanka.

The prevalence of malaria in the country due to indigenous transmission showed a sharp decline in the new millenium, from 210,048 cases in the year 2000 to 0 indigenous cases in 2013 and onward (Figure 3). The proportion of *falciparum* malaria declined from 40% in 2000 to 8% in 2008. While there have been no indigenously transmitted cases of malaria after 2012, a significant number of persons who acquired malaria overseas continued to be identified in the country. Data from the AMC showed that there were 27 imported malaria cases in 2009, 52 in 2010, 51 in 2011, 70 in 2012, 95 in 2013, 49 in 2014, 36 in 2015, 41 in 2016 and 23 in 2017 until mid-May.

The WHO certified Sri Lanka as a malarial-free country in September 2016. This certification was granted to Sri Lanka as there were no indigenous cases of malaria in the preceding 3 years.

A century of efforts by the AMC had finally paid off. However, there is a recognition that careful monitoring needs to be continued with active and passive case detections, and entomological, and parasitological surveillance. It has become particularly important to monitor visitors and travelers from other malaria-endemic countries in view of the regular detection of cases of imported malaria (23). For example, in 2013, imported cases of *P. falciparum malaria* were observed among Sri Lankan fishermen who had returned from working in fishing trawlers in Sierra Leone, while *P. vivax* was detected in a group of asylum seekers from Pakistan (23). Rigorous case detection, effective drug treatment, and long-term monitoring of patients and contacts was considered essential to prevent imported malaria from being transmitted to the indigenous population (23). The importance of active case detection was emphasized from the experience of controlling imported malaria and from observations made on the local population during the pre-elimination phase (24). An unusual case of imported malaria was the detection of the zoonotic parasite *Plasmodium knowlesi*-caused malaria in a soldier returning from peninsula Malaysia (25) where knowlesi malaria is quite common (1). However, the identification of the parasite could only be done in Singapore because local expertise and facilities were not available (24). Because related monkey hosts and anopheline vectors are present in Sri Lanka (1, 26), there is a need to develop local expertise in identifying zoonotic malaria parasites, among which *P. knowlesi*, and to a lesser extent, *Plasmodium cynomolgi*, are presently the most relevant to human health in the Southeast Asian region (1). In addressing the issue of imported malaria, the likely introduction of malaria to Polonnaruwa through importation in the thirteenth century has to be borne in mind.

The following are some essential measures that are being continued for keeping the country free from malaria:

(i) Mosquito vector control measures (these can also reduce the transmission of other endemic mosquito-borne diseases like dengue and chikungunya), e.g., use of treated and untreated mosquito nets, indoor spraying of residual insecticides, as well as clearing blocked drains, filling and ensuring free flow of small streams and rivers to reduce the stagnant water that provides larval habitats. The AMC has recently expanded its mandate to include the control of *Aedes aegypti* and *Aedes albopictus* vectors of dengue and chikungunya, two diseases that have become significant health problems in the country.
(ii) Education of medical staff and the general public about malaria and the dangers of importation from other countries.
(iii) Effective surveillance for and prompt treatment of all cases of imported malaria

## Relevant Features to Consider for Eliminating Malaria in Other Countries

There are several characteristic features that contributed progressively to help eliminate malaria from Sri Lanka in 2013. These are:

(i) Sri Lanka is an island separated by at least 64 km from mainland India. Therefore, infected vectors are not likely to traverse this distance. Cross-border travel is mostly restricted to seaports and airports where good records can be maintained. Malaria control measures applied in the island do not need to consider people moving frequently through unpoliced land borders. This situation is unlike that prevailing in the highly malaria endemic countries of continental Africa and many parts of Asia and South America.
(ii) While high rates of malaria transmission have occurred in localized areas within Sri Lanka, e.g., in areas of large irrigation development, the rates in most parts of the country were often significantly lower than the rates observed in highly malaria endemic countries of sub-Saharan Africa or the Mekong delta (10, 27). Anopheline species with diverse vector potential, ecology, and climate are some factors that may be responsible for the differences in transmission rates (28).
(iii) Sri Lanka is a relatively small country (65,525 km^2^) and has had good educational levels and health facilities for several decades. Government medical clinics and hospitals have been widely present throughout the island for many decades. They are easily accessible and provide free health care to everyone. Therefore, patients with malaria are able to seek medical treatment promptly and have effective antimalaria drugs administered quickly enough to reduce morbidity, mortality, and onward transmission to mosquito vectors (29, 30).
(iv) South American countries have the zoonotic parasites *Plasmodium brasilianum* and *Plasmodium simium* in New World monkeys, and the evidence suggests that these may in fact be *Plasmodium malariae* and *P. vivax*, respectively, which are transmitted both ways between humans and monkeys (1). Other examples of present day zoonosis are *P. vivax*-like parasites in African apes and *P. knowlesi* and more rarely *P. cynomolgi* in Southeast Asia (1). *P. cynomolgi* is found in macaques in Sri Lanka but natural transmission to humans has yet to be documented in Sri Lanka. The occurrence of extensive primate reservoirs of zoonotic malaria parasites makes the elimination of human malaria more difficult, but indigenous zoonotic malaria has not yet been detected in Sri Lanka.
(v) The AMC tasked with controlling malaria was established in 1911, a century prior to the elimination of malaria from the country in 2013. The organization had over this time developed appropriate traditions, protocols and expertise (in entomology, public health, laboratory diagnosis, etc.) needed for controlling malaria and its subsequent elimination. The decentralization of AMC activities to provincial and district levels also allowed greater adaptation to locality-specific needs. The careful use of pyrethroid insecticides by the AMC for indoor residual spraying in most areas after 1993 and in all areas after 2009 would appear to have been particularly effective in reducing malaria transmission.
(vi) The government prioritized and allocated a large budget (at times, about 10% of the total health budget) for malaria control.
(vii) There was considerable commitment on the part of AMC personnel to ensure effective malaria control throughout the island, and equally, the population was for most part receptive to measures such as indoor residual spraying of insecticides and the use of bed nets, mosquito coils, and mosquito repellents to reduce human-vector contact. Malaria control activities were disrupted to varying extent but never entirely stopped during the civil war (1983–2009) in the country. Corruption in officialdom was never an issue in malaria control activities.
(viii) Sri Lankan academics carried out important research pertinent to improving malaria control and actively disseminated research findings within the scientific community and the AMC (10, 31). Recent fundamental scientific discoveries made in Sri Lanka that encouraged local malaria control efforts include: (a) formation of natural transmission-enhancing antibodies in vivax malaria (32), (b) greater malaria transmission as a result of vector population changes in the Mahaweli irrigation scheme (6, 33), (c) presence of *An. culicifacies* sibling species in the country with differential vector potential (34), (d) identification of salinity-tolerant and potent malaria vector *Anopheles sundaicus* in coastal areas (35), and (e) one of the first clinical trials of a malaria vaccine (36).
(ix) Simple educational booklets on malaria written in English and the two vernacular languages of Sinhalese and Tamil were produced by academics and physicians to promote the public understanding of malaria and its control (11). The medical community with non-governmental and voluntary support also organized frequent health camps in remote locations in malaria endemic areas, which resulted in the active detection of malaria cases and their prompt treatment. The health camps also helped raise awareness of malaria in many isolated locations.
(x) Rapid development led to improved housing, communication, transport, and health-care facilities in the country. Greater prosperity also resulted in more widespread use of personal measures that protect against malaria infection, e.g., pyrethroid insecticide sprays and mosquito coils, bed nets, mosquito proofing of houses, and electric fans.

A cross-survey analysis of malaria elimination programs in nine countries (including Sri Lanka) in 2013/2014 concluded that “Political commitment and sustained financing contributed to malaria program success. Consistency of malaria programs depends on political commitment, human and financial resources, and leadership. Operational capacity of the program and the overall health system structure and strength are also important aspects” (37). Countries that have been identified by the WHO to have an increase in either malaria mortality or incidence in the period from 2010 to 2015 are: Nicaragua, Peru, Venezuela, Djibouti, Somalia, Gabon, Kenya, Madagascar, Mali, Namibia, Rwanda, and South Africa (2). These countries go against the general global trend of falling malaria incidence and mortality and appear not have the requisite critical features identified above for successful malaria control (37). They are also not small islands like Sri Lanka and are, therefore, exposed to cross-border and cross-region transmission of malaria. In many of the above countries as well as others showing greater progress with malaria control, cross-border and transnational coordination of malaria control activities and strategies is likely to prove important for eliminating malaria.

## Author Contributions

DAW and RR jointly wrote the manuscript.

## Conflict of Interest Statement

DAW has no conflict of interest to disclose. RR is affiliated to ID-FISH Technology, which has no commercial interest in this article.

## References

[1] Ramasamy R Zoonotic malaria – global overview and research and policy needs. Front Public Health (2014) 2:123 10.3389/fpubh.2014.00123 25184118 PMC4135302

[2] World Health Organization. World Malaria Report 2016. Genève, Switzerland (2016). Available from: http://apps.who.int/iris/bitstream/10665/252038/1/9789241511711-eng.pdf?ua=

[3] World Health Organization. Genève, Switzerland: World Health Organization (2015). Available from: http://www.who.int/malaria/areas/global_technical_strategy/en/

[4] United Nations. New York, USA: United Nations (2015). Available from: http://www.un.org/sustainabledevelopment/sustainable-development-goals/

[5] Ramasamy R Ramasamy MS Wijesundera DA Dewit I Wijesundera A Pathirana S High seasonal malaria transmission rates in the intermediate rainfall zone of Sri Lanka. Ann Trop Med Parasitol (1992) 86:591–600. 10.1080/00034983.1992.11812714 1304700

[6] Ramasamy R De Alwis R Wijesundera A Ramasamy MS Malaria transmission in a new irrigation scheme in Sri Lanka: the emergence of *Anopheles annularis* as a major vector. Am J Trop Med Hyg (1992) 47:547–53. 10.4269/ajtmh.1992.47.547 1449195

[7] Nagendran K Wijesundera DA Wijesundera A Ramasamy MS Ramasamy R Malaria during the 1991-1992 North-East monsoon season in a village in the intermediate rainfall zone of Sri Lanka. J Natl Sci Coun (Sri Lanka) (1993) 21:271–80. 10.4038/jnsfsr.v21i2.8112

[8] Ramasamy R Nagendran K Ramasamy MS Antibodies to epitopes on merozoite and sporozoite surface antigens as serological markers of malaria transmission – studies at a site in the dry zone of Sri Lanka. Am J Trop Med Hyg (1994) 50:537–47. 10.4269/ajtmh.1994.50.537 7515593

[9] Ramasamy MS Kulasekera R Srikrishnaraj KA Ramasamy R Population dynamics of anthropophillic mosquitoes during the northeast monsoon season in the malaria epidemic zone of Sri Lanka. Med Vet Entomol (1994) 8:265–74. 10.1111/j.1365-2915.1994.tb00508.x 7949318

[10] Konradsen F Amerasinghe FP van der Hoek W Amerasinghe PH Malaria in Sri Lanka – Current Knowledge on Transmission and Control. Colombo, Sri Lanka: International Water Management Institute (2000). 77 p.

[11] Ramasamy R Ramasamy MS Wijesundera DA Wijesundera A Malaria and Its Prevention. Kandy, Sri Lanka: Institute of Fundamental Studies (1994). 31 p.

[12] Paranavithana S In: Ray HC, editor. History of Ceylon. Colombo, Sri Lanka: University of Ceylon (1960).

[13] Karunaweera ND Galappaththy GN Wirth DF. On the road to eliminate malaria in Sri Lanka: lessons from history, challenges, gaps in knowledge and research needs. Malar J (2014) 13:59. 10.1186/1475-2875-13-59 24548783 PMC3943480

[14] Wickremasinghe MB Malaria and its control in Sri Lanka. Ceylon Med J (1981) 26:107–15. 6764384

[15] Briercliffe R Dalrymple-Champneys W Discussion on the malaria epidemic in Ceylon 1934-1935. Proc Roy Soc Med (1935) 24:537–62.

[16] Wickremasinghe MB Entomological contributions and needs for malaria control in Sri Lanka. In: Ramasamy R, editor. Current Status of Malaria Research in Sri Lanka. Kandy, Sri Lanka: Institute of Fundamental Studies (1991). p. 38–45.

[17] Abeyasinghe RR Galappaththy GNL Smith Gueye C Kahn JG Feachem RGA. Malaria control and elimination in Sri Lanka: documenting progress and success factors in a conflict setting. PLoS One (2012) 7(8):e43162. 10.1371/journal.pone.0043162 22952642 PMC3430652

[18] Wijesundera DA Malaria, the scourge of Polonnaruwa. Ceylon Med J (1989) 34:113–24. 2695260

[19] Wijesundera DA Wijesundera A Malaria in pregnancy. Contemp Rev Obstet Gynaecol (1994) 29:135–45.

[20] Wijesundere DA Guillain-Barre syndrome in *Plasmodium falciparum* malaria. Postgrad Med J (1992) 68:376–7. 10.1136/pgmj.68.799.376 1630986 PMC2399421

[21] Surendran SN Gajapathy K Kumaran V Tharmatha T Jude PJ Ramasamy R. Molecular evidence for the presence of malaria vector species A of the *Anopheles annularis* complex in Sri Lanka. Parasit Vectors (2011) 4:239. 10.1186/1756-3305-4-239 22192337 PMC3293028

[22] Surendran SN Jude PJ Weerarathne TC Karunaratne SHPP Ramasamy R Variations in susceptibility to common insecticides and resistance mechanisms among morphologically identified sibling species of the malaria vector *Anopheles subpictus* in Sri Lanka. Parasit Vectors (2012) 5:34 10.1186/1756-3305-5-34 22325737 PMC3317438

[23] Dharmawardena P Premaratne RG Gunasekera WM Hewawitarane M Mendis K Fernando D. Characterization of imported malaria, the largest threat to sustained malaria elimination from Sri Lanka. Malar J (2015) 14:177. 10.1186/s12936-015-0697-0 25902716 PMC4411700

[24] Wickremasinghe R Fernando SD Thillekaratne J Wijeyaratne PM Wickremasinghe AR. Importance of active case detection in a malaria elimination programme. Malar J (2014) 13:186. 10.1186/1475-2875-13-186 24885972 PMC4042136

[25] Ranaweera AD Danansuriya MN Pahalagedera K Kumudunayana WM de A W Gunasekera WM Dharmawardena P Diagnostic challenges and case management of the first imported case of *Plasmodium knowlesi* in Sri Lanka. Malar J (2017) 16:126. 10.1186/s12936-017-1776-1 28327145 PMC5361730

[26] Moyes CL Henry AJ Golding N Huang Z Singh B Baird JK Defining the geographical range of the *Plasmodium knowlesi* reservoir. PLoS Negl Trop Dis (2014) 8(3):e2780. 10.1371/journal.pntd.0002780 24676231 PMC3967999

[27] Ramasamy R Malaria transmission levels in Sri Lanka compared to Africa. Ceylon Med J (1992) 37:105. 1291134

[28] Ramasamy R Surendran SN. Global climate change and its potential impact on disease transmission by salinity-tolerant mosquito vectors in coastal zones. Front Physiol (2012) 3:198. 10.3389/fphys.2012.00198 22723781 PMC3377959

[29] Ramasamy R Early treatment of malaria in Sri Lanka and the implications for epidemiology. Ceylon Med J (1992) 37:36. 1581995

[30] Ramasamy R Subanesan N Wijesundera A Fernando NK Ramasamy MS Observations on malaria patients seeking treatment in a rural and an urban hospital in Sri Lanka. J Vector Borne Dis (1992) 29:29–34. 1459297

[31] Ramasamy R, editor. Current Status of Malaria Research in Sri Lanka. Kandy, Sri Lanka: Institute of Fundamental Studies (1990). 106 p.

[32] Peiris JS Premawansa S Ranawaka MB Udagama PV Munasinghe YD Nanayakkara MV Monoclonal and polyclonal antibodies both block and enhance transmission of human *Plasmodium vivax* malaria. Am J Trop Med Hyg (1988) 39:26–32. 10.4269/ajtmh.1988.39.26 3041855

[33] Amerasinghe FP Indrajith NG Post-irrigation breeding patterns of surface water mosquitoes in the Mahaweli Project, Sri Lanka, and comparisons with preceding developmental phases. J Med Entomol (1994) 31:516–23. 10.1093/jmedent/31.4.516 7932596

[34] Surendran SN Ramasamy MS De Silva BG Ramasamy R *Anopheles culicifacies* sibling species B and E in Sri Lanka differ in longevity and in their susceptibility to malaria parasite infection and common insecticides. Med Vet Entomol (2006) 20:153–6. 10.1111/j.1365-2915.2006.00601.x 16608500

[35] Surendran SN Singh OP Jude PJ Ramasamy R Genetic evidence for malaria vectors of the *Anopheles sundaicus* complex in Sri Lanka with morphological characteristics attributed to *Anopheles subpictus* species B. Malar J (2010) 9:343 10.1186/1475-2875-9-343 21114832 PMC3009661

[36] Ramasamy R Wijesundere DA Nagendran K Ramasamy MS Antibody and clinical responses in volunteers to immunization with malaria peptide-diphtheria toxoid conjugates. Clin Exp Immunol (1995) 99:168–74. 10.1111/j.1365-2249.1995.tb05528.x 7851007 PMC1534310

[37] Smith Gueye C Newby G Tulloch J Slutsker L Tanner M Gosling RD. The central role of national programme management for the achievement of malaria elimination: a cross case-study analysis of nine malaria programmes. Malar J (2016) 15:488. 10.1186/s12936-016-1518-9 27659770 PMC5034437

